# An Unusual Case of Pinna Squamous Cell Carcinoma Arising Shortly after Resection of Atypical Fibroxanthoma

**DOI:** 10.1155/2018/6092169

**Published:** 2018-01-23

**Authors:** Eugene Omakobia, Ahmad Orabi, Richard Knights

**Affiliations:** ^1^Department of ENT, Calderdale Royal Hospital, Halifax, UK; ^2^Department of Histopathology, Calderdale Royal Hospital, Halifax, UK

## Abstract

**Objective:**

To report a unique case of pinna squamous cell carcinoma (SCC) arising shortly after resection of atypical fibroxanthoma (AFX) at the same site.

**Case report:**

An 81-year-old gentleman presented with a nodular right pinna lesion. This was excised, and histology confirmed AFX. Ten weeks later, an ulcerative lesion appeared at the resection site. This was confirmed to be SCC. Comparative analysis revealed no morphological resemblance between the initial AFX and new SCC lesion, and there was no evidence of initial misdiagnosis.

**Conclusion:**

SCC is the most common cancer involving the pinna. Whilst prolonged sun exposure is an important risk factor for SCC, chronic inflammation and wounds are other potential sources. We postulate whether SCC could have arisen from the previous scar tissue in a manner similar to Marjolin's ulcer. This would be a highly unusual finding in the pinna and to our knowledge unprecedented in the English literature.

## 1. Introduction

Atypical fibroxanthoma (AFX) is a rare, superficial fibrohistiocytic skin tumour composed predominantly of fusiform, epithelioid, spindle, and pleomorphic cells [1]. It is usually regarded as a benign tumour associated with a good prognosis, but rarely, recurrence and metastasis can occur [2]. It has been suggested that tumours with adverse histological features or extension into subcutaneous fat have a low-to-intermediate malignant potential and should be reclassified as pleomorphic dermal sarcomas rather than AFX [3]. AFX classically presents as a solitary ulcerated nodule on the sun-damaged areas of the head and neck of elderly adults. The pathological differential diagnosis includes pleomorphic dermal sarcoma, spindle cell squamous cell carcinoma, malignant melanoma, and leiomyosarcoma [4].

Poorly differentiated spindle cell neoplasms pose a diagnostic challenge to histopathologists because of a lack of specific morphologic features on standard histopathology. Further characterisation with immunohistochemical studies is always required [5]. AFX can be a particularly difficult diagnosis to make as there are no common guidelines for diagnosis and management [3]. We present a unique case of moderately differentiated squamous cell carcinoma (SCC) arising at the resection site of an AFX of the right pinna. To our knowledge, this is the first reported case of this type in the English literature.

## 2. Case Report

An 81-year-old gentleman presented to our ENT department with a two-year-history of a nodular right pinna lesion. There had been no recent increase in size. The patient reported that the lesion had actually decreased in size over the past two months with the use of topical steroids and fusidic acid.

His past medical history included hypertension, transient ischaemic attack, and prostate malignancy treated with transurethral resection. He also wore bilateral hearing aids for presbyacusis. His regular medications included clopidogrel, simvastatin, and antihypertensives. There was no family history of head and neck malignancy. He was a retired police officer with no history of excessive sun exposure. He was an ex-smoker having stopped smoking 40 years ago and a teetotaller.

**Figure 1:**
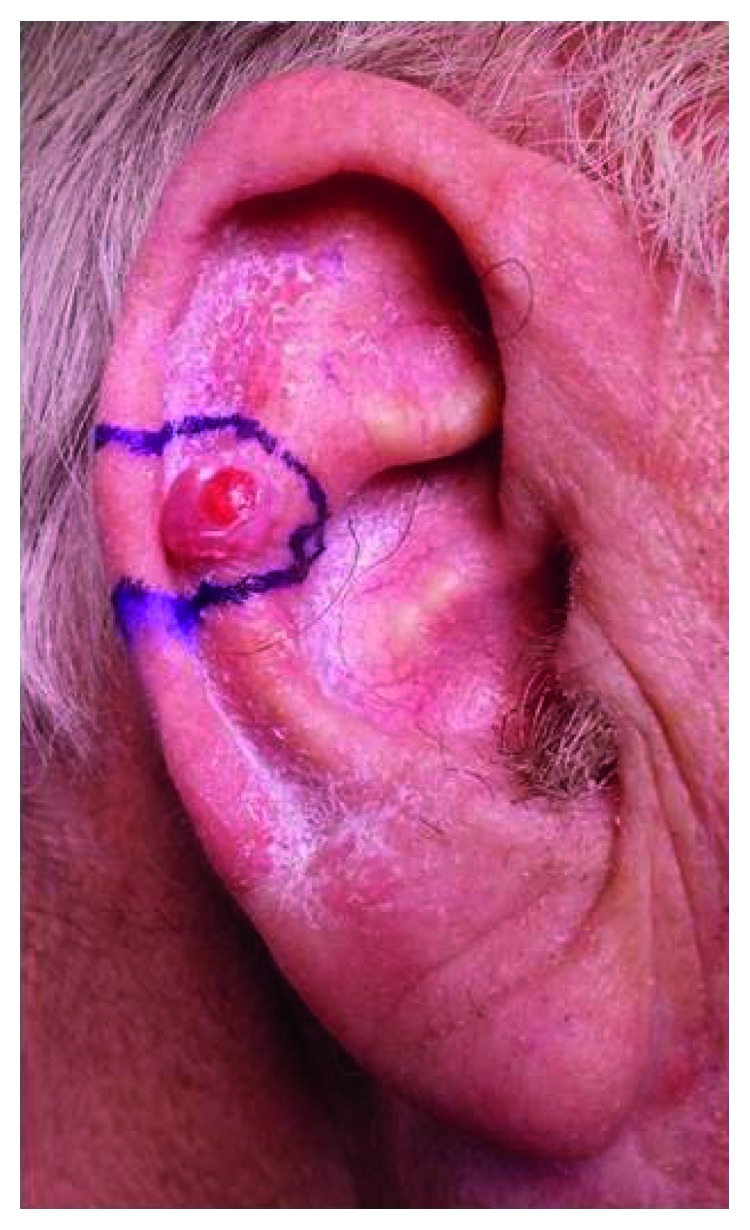
Clinical photograph showing a one-centimetre nodular lesion on the right upper pinna.

On examination, he was skin type II on the Fitzpatrick scale. There was a one-centimetre nodular lesion on the right upper pinna (Figure 1). There was no evidence of ulceration or associated cervical lymphadenopathy, and no other skin lesions were noted. The lesion was not tender to palpation, but there was a history of contact bleeding after irritation from his hearing aid.

**Figure 2:**
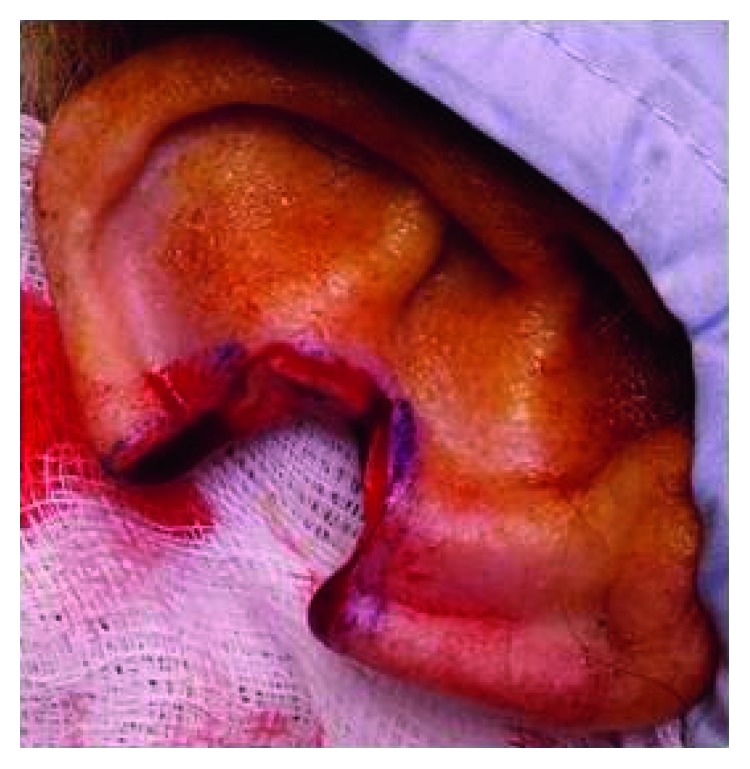
Clinical photograph showing appearance of the right upper pinna following wedge excision of the initial one-centimetre nodular lesion with 4-millimetre resection margins.

**Figure 3:**
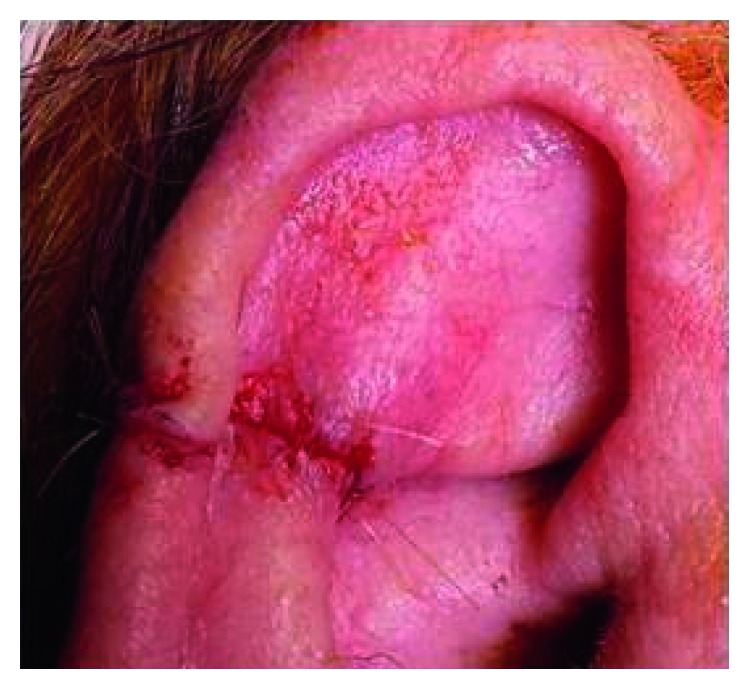
Clinical photograph showing right pinna after wedge excision of the initial nodular lesion and primary wound closure.

**Figure 4:**
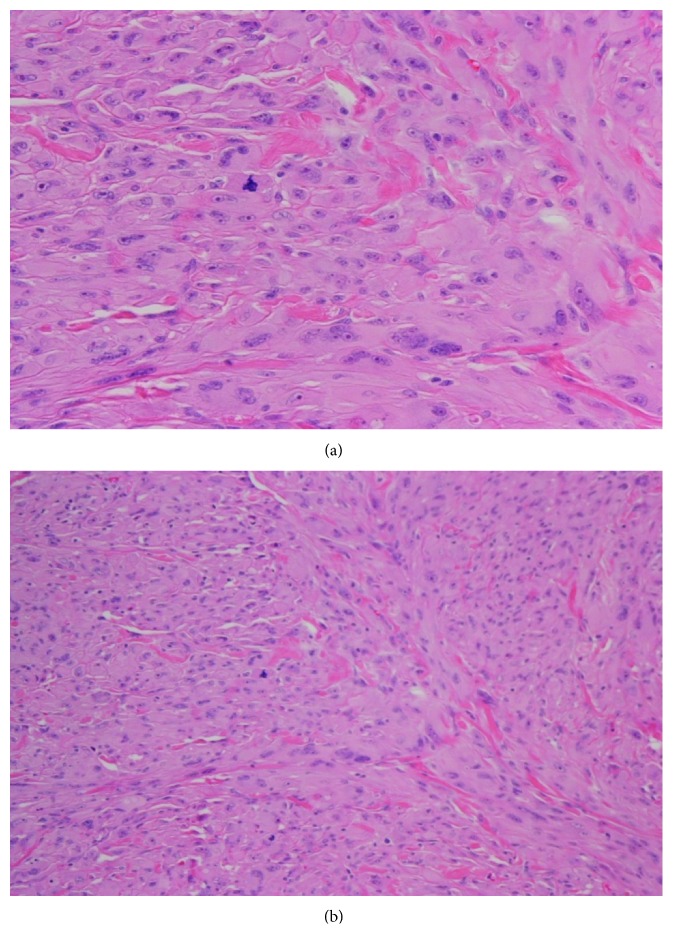
Photomicrograph of excised nodular lesion (H&E stain) showing a dermal spindle cell lesion exhibiting marked pleomorphism and brisk mitoses. Immunohistochemically, focal staining with CD68 was seen. Morphologically and immunohistochemically, the features are consistent with AFX. (a) ×40 magnification. (b) ×20 magnification.

Two weeks after initial presentation, a wedge excision of the lesion was performed under local anaesthesia with 4-millimetre resection margins (Figures 2 and 3). Histological analysis revealed a dermal spindle cell lesion exhibiting marked pleomorphism and brisk mitoses (Figure 4). Immunohistochemically, the lesion showed focal staining with CD68. Other markers (AE1AE3, S100, HMB45, SMA, and CD31/34) were negative. Morphologically and immunohistochemically, the features were consistent with atypical fibroxanthoma. The closest resection margin was found to be 3 millimetres located superiorly. Resected sleeves of cartilage at the inferior and superior margins were found to be free of disease.

**Figure 5:**
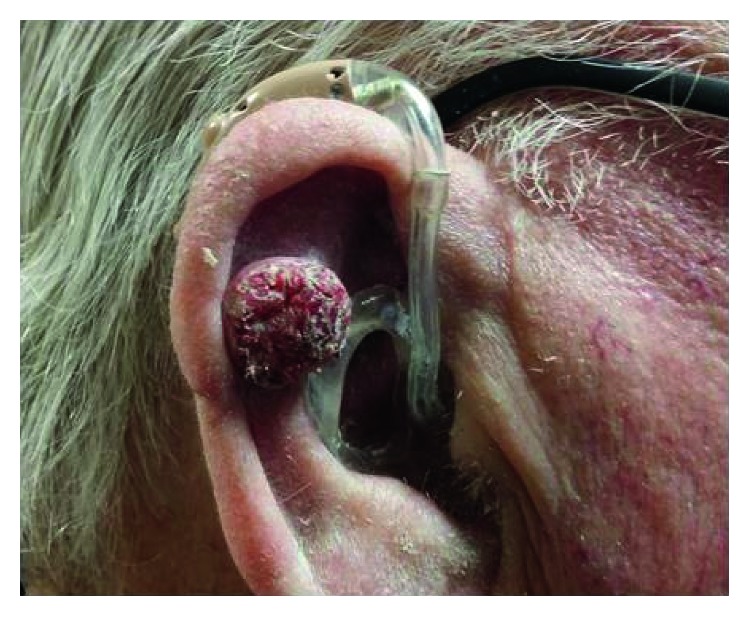
Clinical photograph showing the right pinna with a one-centimetre raised, ulcerative, crusted lesion at the upper margin of the previous incision site.

**Figure 6:**
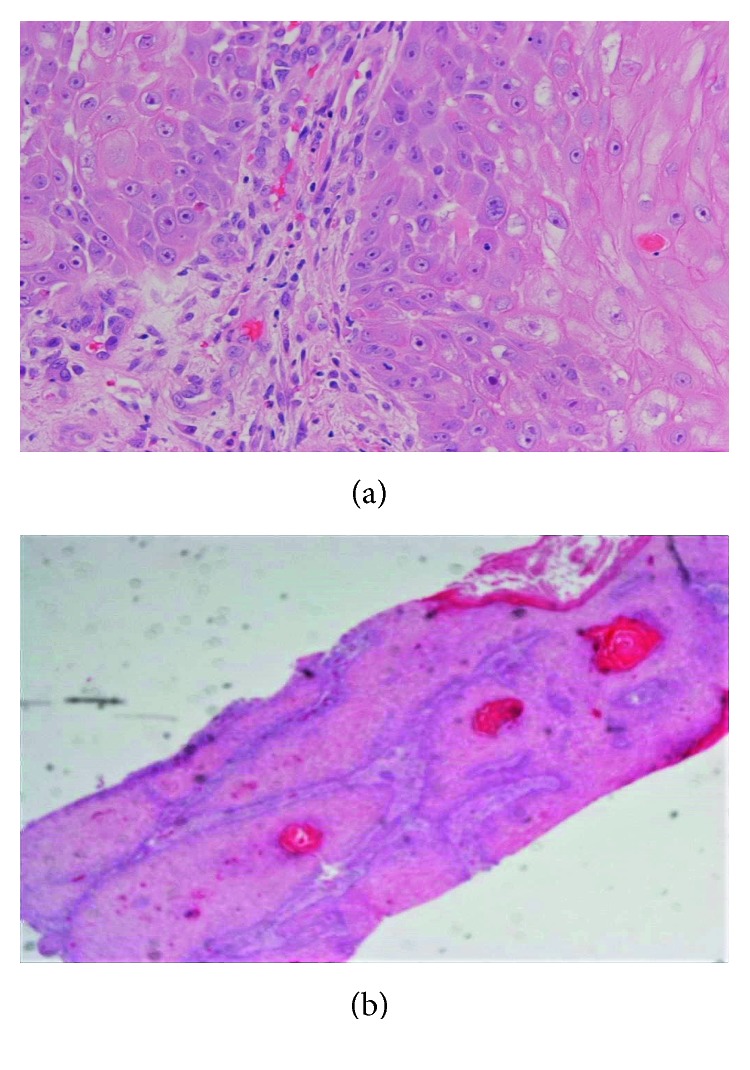
Photomicrograph of punch biopsy from the second ulcerative lesion (H&E stain). This demonstrates full-thickness infiltration by a well-differentiated keratinising SCC. (a) ×20 magnification. (b) ×2.5 magnification.

One month later, a small fluctuant lesion was noted at the surgical wound site laterally. This was felt to be a small stitch abscess, but needle aspiration yielded no fluid. A decision was made to simply observe this, and the patient was brought back for review six weeks later. At this time, a one-centimetre raised, ulcerative, crusted lesion was seen at the upper margin of the incision site (Figure 5). Three-millimetre punch biopsies were taken which were subsequently discussed at the skin multidisciplinary team (MDT) meeting. The histological analysis showed full-thickness infiltration by a well-differentiated keratinising squamous cell carcinoma (Figure 6). There were no lymphovascular space permeation and no associated squamous hyperplasia. Remarkably, when this tumour was compared to the previous lesion (atypical fibroxanthoma), no morphological resemblance was seen. Immunohistochemistry of the biopsy confirmed squamous cell carcinoma (positive with AE1AE3 and p63 and negative with CD10).

**Figure 7:**
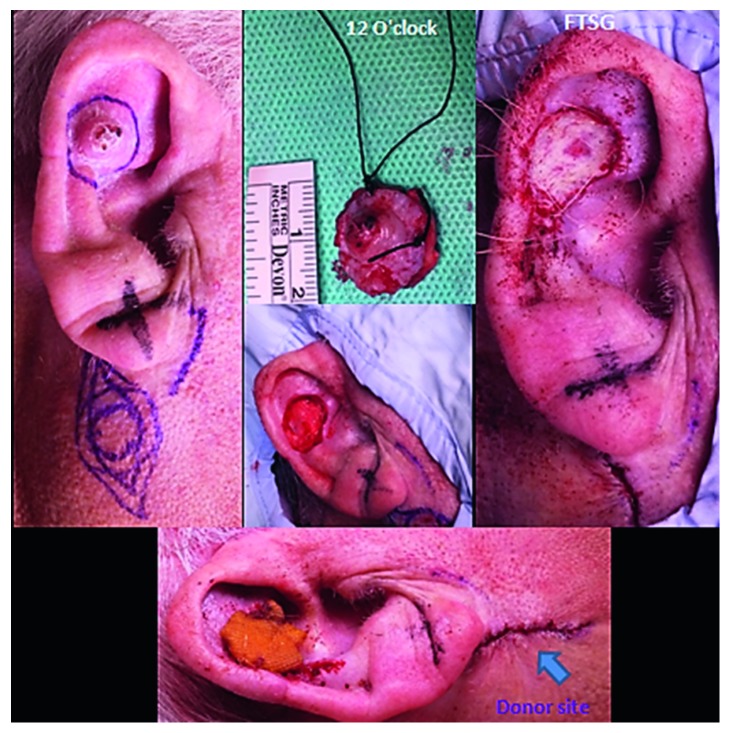
Clinical photographs showing wide excision (with 5-millimetre resection margins) of the right pinna ulcerative, crusted lesion and full-thickness skin grafting (FTSG). The underlying cartilage has also been excised, and a local full-thickness skin graft was raised from the right level II region to cover the skin defect. Primary closure of the donor site is also illustrated in the lower image.

**Figure 8:**
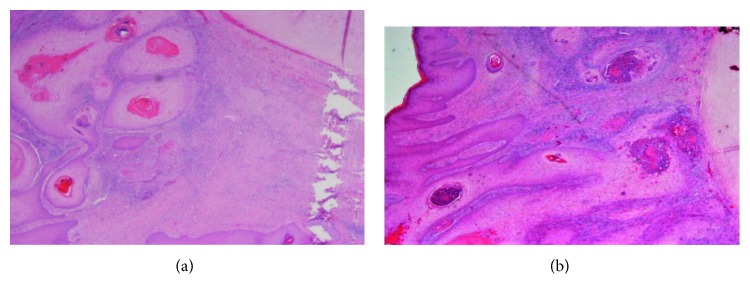
Photomicrograph of excised ulcerative, crusted lesion (H&E stain). This demonstrates moderately differentiated SCC. (a) ×10 magnification. (b) ×2.5 magnification.

**Figure 9:**
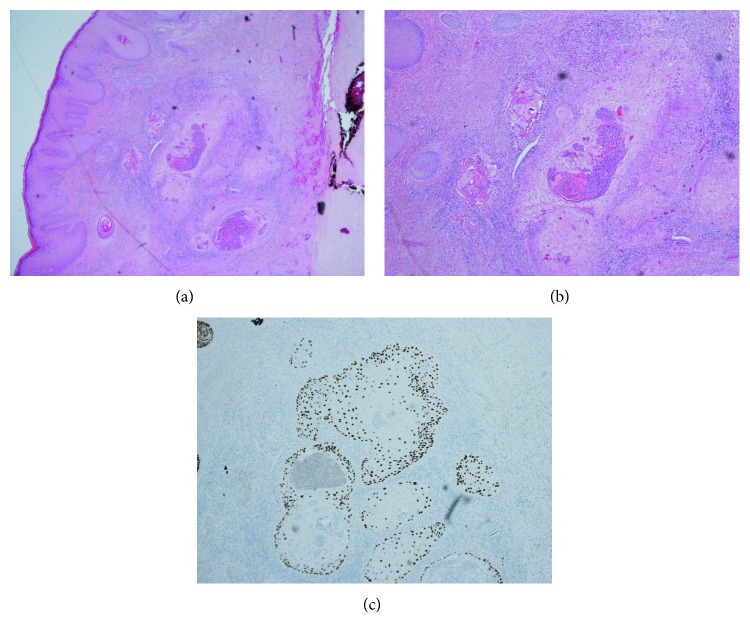
(a) Photomicrograph of wide locally excised ulcerative, crusted lesion (H&E stain) at ×2 magnification. Nests of squamous cell carcinoma are seen infiltrating the dermal tissue without involvement of the underlying cartilage (cartilage is seen in the right-hand field). (b) ×4 magnification of the same area; this demonstrates infiltrating nests of keratinising squamous cell carcinoma associated with a host inflammatory response. (c) p63 immunohistochemistry confirms the squamous phenotype of the tumour (nuclear positivity).

**Figure 10:**
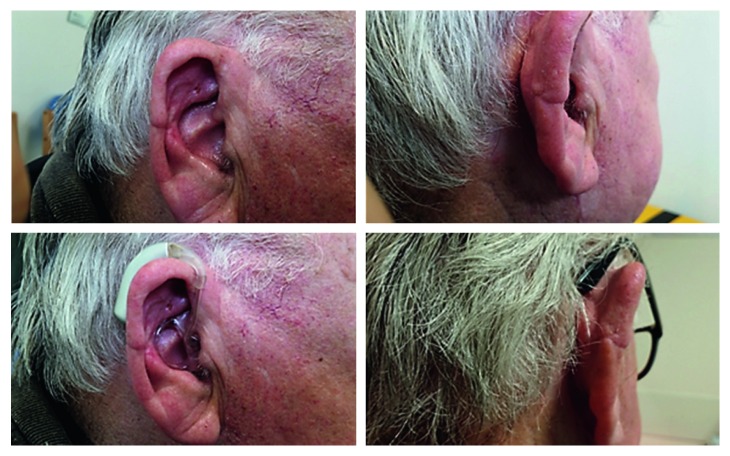
Clinical photographs showing the right pinna appearance at the most recent 4-month follow-up. The wound has healed well with successful grafting. The patient is comfortably able to wear his hearing aids and spectacles.

The recommendation from the skin MDT (multidisciplinary team) was for wide excision and full-thickness skin grafting. This was performed three weeks later with 5-millimetre resection margins. The underlying cartilage was also excised, and a local full-thickness skin graft was raised from the right neck level II region to cover the skin defect (Figure 7). Subsequent histological and immunohistochemical analyses confirmed moderately differentiated squamous cell carcinoma with no invasion of the underlying cartilage or subcutaneous fat. There was no perineural or lymphovascular invasion, and the lesion was 4.5 millimetres thick (Figures 8 and 9(a)–9(c)). The margins were deemed acceptable following further discussion at the skin MDT meeting, and an ultrasound scan of the neck was organised, which showed normal cervical lymph nodes. To date, no local or regional recurrence has been noted at his most recent 4-month follow-up, and the wound has healed well with successful grafting (Figure 10).

## 3. Discussion

We have reported an unusual case of moderately differentiated pinna SCC arising at the resection site of AFX. This is unprecedented in the world literature. We conducted a literature search to gain further insight into how this may have arisen.

It is widely reported that diagnostic difficulties can exist particularly in the distinction between poorly differentiated cutaneous spindle cell squamous cell carcinoma (scSCC) and AFX [3, 5, 6]. This is due to the fact that AFX is generally accepted as a diagnosis of exclusion based on negativity for a broad panel of immunohistochemical markers, including multiple cytokeratins and melanocytic, muscle, and vascular markers [6]. A recent review article has suggested that the most important immunomarkers for differential diagnoses are cytokeratins (to exclude sarcomatoid or spindle cell SCC), S-100 and melanogenesis markers (to exclude melanoma), and desmin, actin, and H-caldesmon (to exclude leiomyosarcoma) [3]. As cytokeratins can also be occasionally lost in scSCC, this can pose diagnostic challenges. However, in our reported case, the second tumour was a moderately differentiated squamous cell carcinoma, which was not of the spindle cell variant, thereby reducing diagnostic uncertainty. Moreover, when the subsequently occurring SCC was compared to the previous AFX lesion, no morphological resemblance was seen.

In cases of poorly differentiated or spindle cell variants of invasive SCC, immunohistochemical studies for p63 are widely regarded as useful in confirming squamous differentiation, with particular utility in the diagnostic differentiation from AFX and melanoma [7, 8]. Hence, p63 is a useful addition to the standard immunohistochemical panel for cutaneous spindle cell neoplasms. Strong expression of p63 suggests scSCC and argues against the diagnosis of AFX.

AFX is usually a low-risk tumour, but as stated, care must be taken in diagnosis with exclusion of the more aggressive scSCC using immunohistochemical studies. We considered that a misdiagnosis of AFX which has been previously reported [5] may have been related to the subsequent development of SCC. However, in our reported case, the SCC was moderately differentiated and not of a spindle cell pattern. Comparative analysis revealed no similarities between the initial AFX and the new SCC lesion. Initial misdiagnosis is therefore highly unlikely. The case reiterates the importance of always considering a recurrent or new primary tumour at surgical resection sites.

Another factor to be considered is the possibility of metachronous (consecutive) development of AFX and SCC at the same site. This is plausible since UV radiation is known to be the major causative factor for cutaneous SCC, and both clinical and molecular data suggest that UV radiation is also a triggering factor for AFX [4].

We further speculated on whether the SCC arose from the previous scar tissue in a similar fashion to Marjolin's ulcer. Marjolin's ulcer classically occurs following previous trauma to tissue such as chronic inflammation, burns, or within any form of scar tissue, including postsurgical scars. It tends to be a more aggressive form of SCC which is usually well differentiated when biopsied [9]. There is commonly a delay between the initial tissue insult and the development of malignancy. This time period has been defined as latency and ranges between 11 and 75 years [10]. However, there have been reports of “acute” Marjolin's ulcer occurring after months or even weeks [11]. Marjolin's ulcer is not an uncommon finding in the lower limbs but is vanishingly rare in the head and neck site. Indeed, there is only one similar report in the literature of Marjolin's ulcer occurring in the postauricular region nine months after cortical mastoidectomy [11]. However, there are no reports of this pathology occurring in the pinna; hence, this would be a novel case. In the reported case, there was a short 10-week latency period after the initial surgery, making Marjolin's ulcer unlikely but not impossible. Due to the more aggressive nature of these tumours, it is imperative to monitor Marjolin's ulcers for metastasis and recurrence. This is an important management consideration, and as in our case report, we would advocate the inclusion of baseline neck ultrasonography to assess cervical lymph nodes for involvement.

## 4. Conclusion

The finding of pinna SCC arising shortly after resection of AFX is extremely rare and, to our knowledge, unprecedented in the world literature. This finding does not appear to be related to an initial misdiagnosis of AFX. It is conceivable that the SCC could represent a metachronous tumour or a form of Marjolin's ulcer. Whilst this has been previously reported in the postauricular region, it has never been documented at the site of the pinna, making this a highly intriguing clinical presentation.

## Additional Points

- Poorly differentiated spindle cell neoplasms pose a diagnostic challenge to histopathologists.
- AFX is usually a low-risk tumour, but care must be taken in diagnosis with exclusion of the more aggressive spindle cell SCC using immunohistochemical studies.
- It is crucial to consider a recurrent or new primary tumour at surgical resection sites.
- SCC could have arisen from previous scar tissue in a similar fashion to Marjolin's ulcer, although this has not been previously reported in the pinna.
- Due to the more aggressive nature of these tumours, we would advocate the inclusion of baseline neck ultrasonography to assess cervical lymph nodal involvement.
